# Fabrication and Optimization of High Aspect Ratio Through-Silicon-Vias Electroplating for 3D Inductor

**DOI:** 10.3390/mi9100528

**Published:** 2018-10-18

**Authors:** Haiwang Li, Jiasi Liu, Tiantong Xu, Jingchao Xia, Xiao Tan, Zhi Tao

**Affiliations:** National Key Laboratory of Science and Technology on Aero Engines Aero-Thermodynamics, Beihang University, Beijing 100191, China; 19820912@sina.com (H.L.); jiasi0820@163.com (J.L.); christxtt@163.com (T.X.); jingchaoX@buaa.edu.cn (J.X.); by1404143@buaa.edu.cn (X.T.)

**Keywords:** through-silicon-vias (TSV), high aspect ratio, control variable method, electroplating, three-dimensional (3D) inductor

## Abstract

In this study, the filling process of high aspect ratio through-silicon-vias (TSVs) under dense conditions using the electroplating method was efficiently achieved and optimized. Pulsed power was used as the experimental power source and the electroplating solution was prepared with various additive concentrations. Designed control variable experiments were conducted to determine the optimized method. In the control variable experiments, the relationship of multiple experimental variables, including current density (0.25–2 A/dm^2^), additive concentration (0.5–2 mL/L), and different shapes of TSVs (circle, oral, and square), were systematically analyzed. Considering the electroplating speed and quality, the influence of different factors on experimental results and the optimized parameters were determined. The results showed that increasing current density improved the electroplating speed but decreased the quality. Additives worked well, whereas their concentrations were controlled within a suitable range. The TSV shape also influenced the electroplating result. When the current density was 1.5 A/dm^2^ and the additive concentration was 1 mL/L, the TSV filling was relatively better. With the optimized parameters, 500-μm-deep TSVs with a high aspect ratio of 10:1 were fully filled in 20 h, and the via density reached 70/mm^2^. Finally, optimized parameters were adopted, and the electroplating of 1000-μm-deep TSVs with a diameter of 100 μm was completed in 45 h, which is the deepest and smallest through which a three-dimensional inductor has ever been successfully fabricated.

## 1. Introduction

With the requirements of reduced feature size and increased transistor performance for semiconductor technology, three-dimensional (3D) integration technology—using through-silicon-vias (TSVs) to realize the interconnection of multi-stacked chips—has emerged as a promising technology [1,2,3,4]. 3D integration technology can produce shorter interconnections in the vertical direction, resulting in a faster response and better performance of integrated circuits (ICs).

High-aspect-ratio TSVs applied in electronic devices are light, thin, and small, having an important role in MEMS (micro-electro-mechanical system) devices, including MEMS sensors, 3D inductors, micro-motors, and micro-converters [5,6,7]. Since the development trend of miniaturization and improving efficiency for current electric products, increasing TSVs conduction in a smaller area, using high-density TSVs, is necessary [8,9]. High-density TSVs help reduce the size of MEMS devices and increase power density, promoting the development and performance improvement of MEMS devices [10,11].

Copper electroplating is extensively used in high-aspect-ratio TSV interconnections due to the lower resistivity and higher resistance for electromigration, which has been reported in many studies, and has become a relatively mature method [12]. During the electroplating process, several variables, including current density and additives concentration, may significantly impact the electroplating results. However, achieving complete filling in high-aspect-ratio TSVs remains a challenge, and so has become a research focus. Numerous methods have been proposed to achieve full copper filling in high-aspect-ratio TSVs [13,14,15].

Tian et al. [16] realized TSV filling with an aspect ratio of 6:1 using pulse-reverse current electrodeposition and studied the filling performance at different positive and negative currents. Li et al. [17] summarized the effect of different pretreatment methods on through silicon with copper filling. Ultrasound, vacuuming, and wetting treatments with various reagents and other treatments were investigated as pretreatments in their study. Xu et al. [18] adopted a vacuum pretreatment method using methylsulfonate with methane sulfonic acid as the base electroplating solution. They have achieved 370-μm-deep TSV vias with a diameter of 60 μm with complete filling and high density based on bottom-up copper electroplating. Chuang et al. [19] fully filled TSVs with a depth of 525 μm and a high aspect ratio of 7:1. In the current research, the highest aspect ratio of electroplated TSVs reached 10:1 with a depth of 200 μm [20], whereas the TSVs to be electroplated were not high-density in their experiments.

Voids and seams are easily formed during the electroplating process, which result in incomplete filling and serious reliability problems. Ensuring uniformity and fully filling in all TSVs under dense conditions remains a challenge. Under high density conditions, nonuniform growth may cause voids or seams to form in the through vias. To grow electroplated copper uniformly in high-aspect-ratio TSVs under high density conditions, precise control of multiple variables is required.

Therefore, this study focused on the filling process for high density, small diameter, high-aspect-ratio TSVs. We systematically analyzed the experimental variables influencing the electroplating result. Detailed experimental steps, including preparing the wafers to be electroplated, building the electroplating device, pretreatment method, the electroplating experiments, data comparison, and analysis, are reported. Experiments for different variables (current density and additives concentration) were performed to investigate the optimized configuration. Finally, we summarized the optimized variable through complementary experiments and realized complete filling for high-aspect-ratio (10:1) TSVs (500 μm) with the maximum distribution density reaching 70/mm^2^.

## 2. Experimental Design

### 2.1. Copper Filling Mechanism

The composition of the basic electroplating solution used in the TSV filling experiment is shown in Table 1. Additives, which included Cupracid Ultra Make-up, Cupracid Ultra A, and Cupracid Ultra B from Atotech (Berlin, Germany) were added to the basic solution. Cupracid Ultra A and Cupracid Ultra B were used as the accelerator and leveler, respectively, and worked together to obtain an ideal coverage, whose concentration and proportions had to be maintained within a certain range.

The plating pulse power source was a product of Xiamen Qunji (Xiamen, China), which had an output current of 0–2 A (ΔA≤±10 mA,R=1 kΩ), voltage of 0–20 V (ΔV≤±100 mV,I≤±0.5 A). The pulse wavelength ranged from 0.01 to 9999 ms (N ≤24).

**Figure 1:**
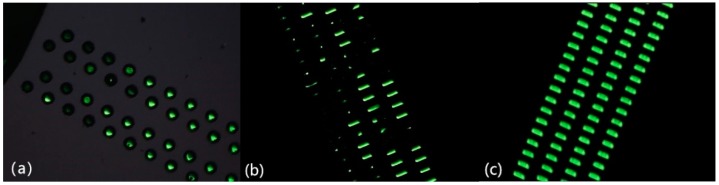
Results of three pretreatment methods observed through the dark field photographed with an optical microscope. (**a**) ultrasonic cleaning, (**b**) rinsing with deionized water, and (**c**) vacuum processing.

Before the electroplating process starting, pretreatment work to remove air bubbles in the vias was required. In general, there are three pretreatment methods: ultrasonic cleaning, rinsing with deionized water, and vacuum processing [21,22]. To determine whether there were bubbles in the TSVs soaked in the electroplated solution, we chose to observe those holes through the dark field of the microscope, from Hitachi (Tokyo, Japan). The light parts indicated that air bubbles have been completely removed, confirming that the TSVs were well prewetted. Dark parts mean air bubbles still existed in the vias, which may impede copper growth. The results of three pretreatment methods are found in Figure 1a–c. Based on those figures, we proved that vacuum processing exerts the best effect on removing the air bubbles since all the TSVs areas were bright.

**Figure 2:**
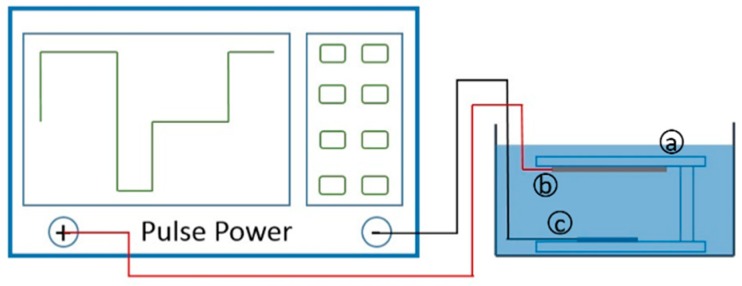
Experimental set-up schematic for through-silicon-vias (TSVs) filling (**a**) Teflon jig (13 cm × 16 cm × 0.5 cm, h = 3 cm); (**b**) copper anode; (**c**) silicon wafer cathode to be electrodeposited (13 mm × 21 mm × 0.5 mm).

The copper electroplating procedure was performed in a rectangular groove (14 cm × 19 cm × 15 cm). The whole electroplating device principle is presented in Figure 2. In the preparation step, the anode copper sheet and cathode silicon wafer were fixed on the Teflon jig and placed in the electroplating solution. The results showed that the use of an anode bag effectively enhances the uniformity of the electroplated copper under the same experimental conditions.

After vacuum pretreatment, the anode copper sheet and cathode silicon wafer with seed layer were connected to the positive and negative poles of the pulse power. Since pulse current produces a better electroplating effect than direct current (DC) [23], we adopted pulse current as the electroplating current source. The time proportion of the positive current to negative current to empty was 60:10:60 ms (6:1:6).

### 2.2. Fabrication of the TSV Sample

The TSV sample fabrication process was divided into four parts: wafer cleaning, formation of high aspect ratio vias, barrier layer deposition, and seed layer deposition. This section explains the processing details.

The 500-μm-thick monocrystalline silicon (Si) wafers with double-sided oxidation were used in the current experiment. First, the surface of the wafer was cleaned with acetone or alcohol and then soaked in deionized (DI) water for 10 min before drying by N_2_. The TSV etching areas were defined and patterned using the lithography process. The positive photoresist (AZ4620, Suzhou, China) was spin-coated on the double-side of the substrate as the protecting layer. Subsequently, the exposure and development process was used to transfer the desired etching pattern from the mask to the photoresist layer. Afterward, we soaked the substrate in buffer oxidized etching (BOE) solution to remove the oxidation layer on the surface due to the low removing speed during the inductively coupled plasma (ICP) etching process.

The through-silicon-vias (50 μm in diameter) were etched using an inductively coupled plasma (ICP) from the SPTS Company (Newport, UK), which generated the source plasma through an inductively coupled coil (1 kW, 13.56 MHz). The etching process was conducted in a low temperature environment, in which helium was supplied to the backside of the wafer as the cooling gas. The whole etching process was divided into an etching step and a passivation step, and sulfur hexafluoride (SF_6_) and octafluoroyclobutane (C_4_F_8_) were used as the required gases. The etching recipe was defined based on the standard Bosch anisotropic etching method. In the etching step, the source power was set to 600 W, bias power was 15 W, the SF_6_ gas flow rate was 130 sccm, the O_2_ gas flow rate was 13 sccm, and the time for the etching step was 8 s. In the passivation step, the source power was set to 600 W, bias power was 0 W, C_4_F_8_ gas flow rate was 85 sccm, and the time was 5 s. The etching rate was approximately 2.3 μm/min.

Since the diameters of through vias are so small that it is difficult to successfully etch only once, the etching process should be performed twice. Once the silicon wafer was etched to a depth of about 250 μm, we stopped etching, repeated the above operation, and etched the same pattern on the opposite side.

**Figure 3:**
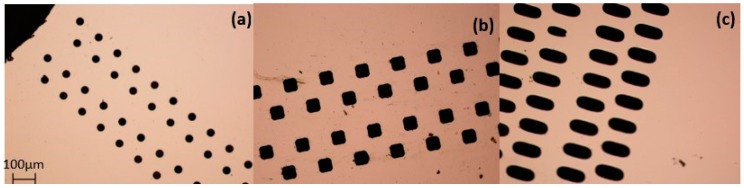
Silicon substrate sample for electroplating, obtained with an optical microscope. (**a**) circle, (**b**) square, and (**c**) oval holes.

After ICP etching, the silicon wafers with through vias were placed in a high temperature furnace to deposit the SiO_2_ insulation layer to prevent the transmission of electrical signals and avoid deposition occurring from the side walls during the electroplating process. Afterward, the adhesion layer (Cr 30 nm) and seed layer (Cu 300 nm) were sputtered on the SiO_2_ layer using a magnetron sputtering machine MSP-300B (Beijing, China). Figure 3 presents the silicon substrate sample after magnetron sputtering.

**Figure 4:**
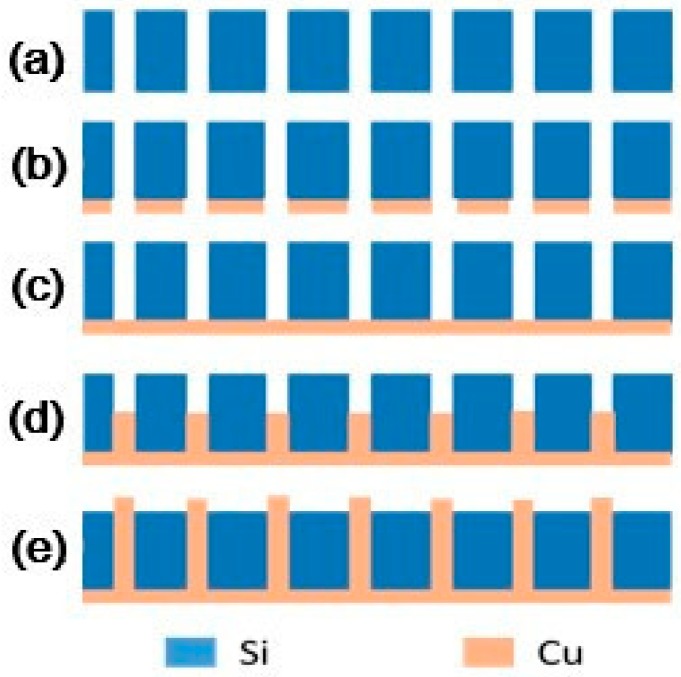
The electroplated copper growth process. (**a**) TSVs were etched and the SiO_2_ insulation layer was deposited. (**b**) The adhesion layer (Cr, 30 nm) and seed layer (Cu, 300 nm) were sputtered. (**c**) Electroplated copper covered the TSVs. (**d**) Electroplated copper was grown in TSVs. (**e**) The TSVs were fully filled.

The bottom-up electroplating method was used in this research so that the seed layer could be used to create a conductive effect during the electroplating process. After completing the above process, the TSV sample fabrication was finished and was followed by the copper electroplating process. The steps for sample preparation and copper growth are shown in Figure 4.

### 2.3. Control Variable Experimental Design

As previously reported [24,25,26], several parameters, like current density and additives concentration, may influence the electroplating results especially for micro-scale through vias. For example, electroplated copper would grow too fast and non-uniformly with excessive current. Insufficient additive concentration may result in low electroplated copper quality, which means the electroplated copper is rough and not bright with uneven growth [27,28]. To find and optimize the electroplating configuration that would relatively quickly and fully fill through silicon vias, we designed a controlling variable experiment based on the mentioned variables.

Controlled-variable experimental methodology is one of the most useful techniques for variance reduction using known information to reduce the error when estimating a suitable variable range. Only one variable is changed at a time, while the remaining factors are kept constant. This method is frequently used for multivariate problems since the multi-variable problem can become a multiple single variable problem. Therefore, we separately studied the influence of changing factors on experimental results.

We chose additive concentration, current density, and different shapes of through vias as three experimental factors. Among these factors, according to their suitable range, the parameters of the controlling variable experimental method were selected, as shown in Table 2.

## 3. Results and Discussion

The growth rate and uniformity of the electroplated copper were detected using microscope observation, from Hitachi (Tokyo, Japan), a scanning electron microscope (SEM), from SEC (Suwon, Gyeonggi-do, South Korea), and a confocal displacement detector (CL3-MG70), from Thinkfocus (Hebei, China) for time. The experimental results are shown in Table 3. To determine the uniformity of copper growth in the vias, we simultaneously used the confocal displacement detector to obtain the maximum height difference of the electroplated copper growth.

### 3.1. Effect of Current Density

**Figure 5:**
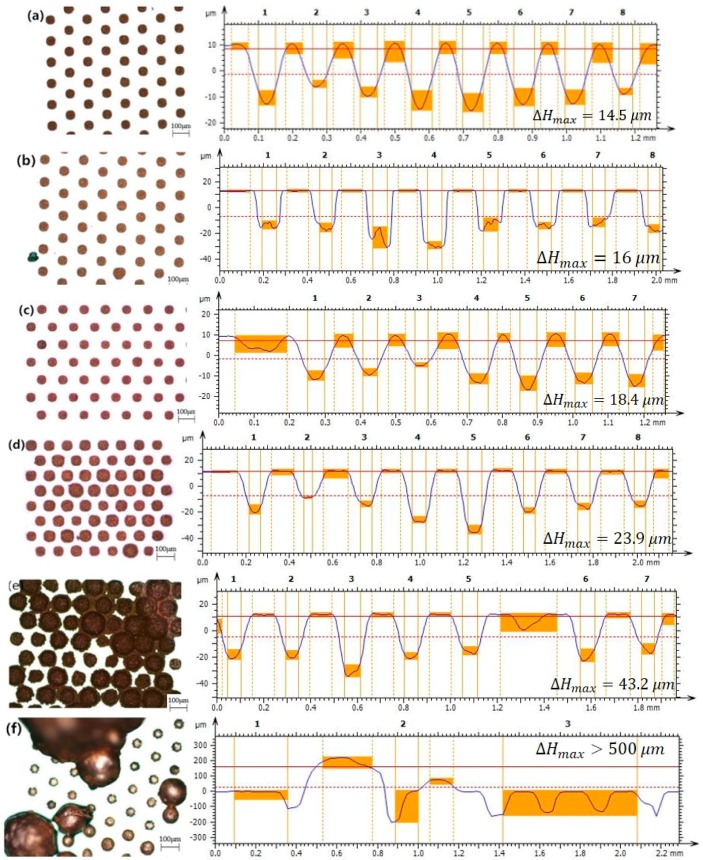
Top views and profile height diagram taken from the section displaying the greatest height difference under different current conditions using an optical microscope and a confocal displacement detector: (**a**) 0.5 A/dm^2^, (**b**) 1 A/dm^2^, (**c**) 1.25 A/dm^2^, (**d**) 1.5 A/dm^2^, (**e**) 1.75 A/dm^2^, and (**f**) 2 A/dm^2^.

Experiments 1 to 7 were designed to determine the relationship between current density and electroplating effect, in which the current density from 0.25 to 2 A/dm^2^ was used for the experimental variables. The top views of the copper filling TSV arrays were photographed using an optical microscope. To obtain the results, we selected the cross-section with the greatest height, analyzed the topographical features of the section, and obtained the maximum height of the section. The top views and profile height diagram obtained from the most disparate section in terms of height difference are shown in Figure 5a–f.

The results show that the TSV bottom parts failed to close because of the tiny current promotion with a current density under 0.25 A/dm^2^ [29] ith increasing current density, the electroplating speed increased approximately linearly with the current density in the range of 0.5 to 1.75 A/dm^2^. However, the electroplating quality gradually decreased.

We measured the distance from the electroplated copper to the silicon wafer surface in each via and calculated the height of each electroplated copper and their coefficient of variation in the region. It could be concluded that the smaller the coefficient of variation, the smaller the difference in growth rate of electroplated copper among different vias, which means better uniformity.

The maximum height difference among the filled through-vias increased up to 23.9 μm under a 1.5 A/dm^2^ current density. Uniform filling of all through vias under high density conditions (70/mm²) was achieved within 20 h. With the current density increasing above 1.5 A/dm^2^, the electroplating speed increased rapidly, while the electroplated copper uniformity decreased sharply since the coefficient of variation increased from 0.19 to 0.26. The current density further increased to 2 A/dm^2^, indicating that complete filling of through-vias could not be achieved. TSVs appeared to be covered by copper grown in other adjacent vias. Considering speed and uniformity together, we concluded that a current density of 1.5 A/dm^2^ produced the best experimental results under the same conditions.

Based on previous experiments [26,27], when the current density is too small, the speed of copper ion transfer is insufficient, which results in a small growing promotion effect of electroplated copper. With the increase in the current density, the electroplating reaction rate improved. Since the Cu^2+^ transferring rate was fast, the ion distribution in the solution was not uniform, which would lead to the failure of the copper full filling.

### 3.2. Effect of Additive Concentration

**Figure 6:**
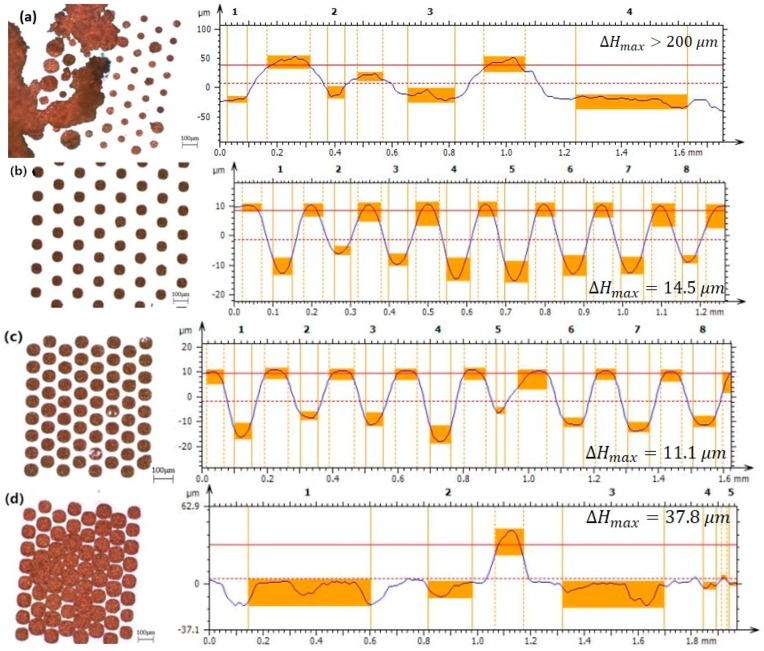
Top views and profile height diagram taken from the section with the greatest height difference under different additive concentrations obtained with an optical microscope and confocal displacement detector: (**a**) A: 0.25 mL/L, B: 0.25 mL/L; (**b**) A: 0.5 mL/L, B: 0.5 mL/L; (**c**) A: 1 mL/L, B: 1 mL/L; and (**d**) A: 1.5 mL/L, B: 1.5 mL/L.

Experiments 3 and 8–10 were designed to investigate the effect of additive concentration on the filling. Variable experiments with several concentrations of additives were selected, ranging from 0.25 to 1.5 mL/L. Experimental parameters and results are shown in Figure 6a–d. We proved that the appropriate additive concentration range is 0.5 to 1 mL/L in high-aspect-ratio TSV filling experiments. No additives or too low a concentration of additives in the electroplating solution would result in rough electroplating and would not fully fill the TSVs. With too high an additive concentration (>1.5 mL/L), the uniformity of the electroplated copper considerably decreased and the anode slime fell off much faster. In the experiments, additives worked together to achieve electroplating copper filling (“hole bottom acceleration, orifice suppression”) by controlling the reaction $\mathrm{Cu}\to {\mathrm{Cu}}^{+}$, ${\mathrm{Cu}}^{+}\to {\mathrm{Cu}}^{2+}$ on the anode. When the concentration was too low, the levelling effect was not achieved. Excessive additives would accelerate the reaction process $\mathrm{Cu}\to {\mathrm{Cu}}^{+}$, possibly causing the anode slime to fall off, affecting the experiment.

### 3.3. Effect of Different TSV Shapes

To determine the effects of different via shapes on the electroplating result, we performed TSV electroplating experiments with three shapes (circle, oval, and square) and compared the results in terms of growing speed and electroplated copper quality under the same experimental condition. In order to determine the experimental current density and additive concentration parameters, we selected values from the appropriate range of current variables and additive variable experiments, and completed the experiments using a current density of 1 ASD and an additive concentration of 1 mL/L. The through-vias of different shapes had the same sectional area, equal to the area of a 100-μm-diameter circle through-via.

**Figure 7:**
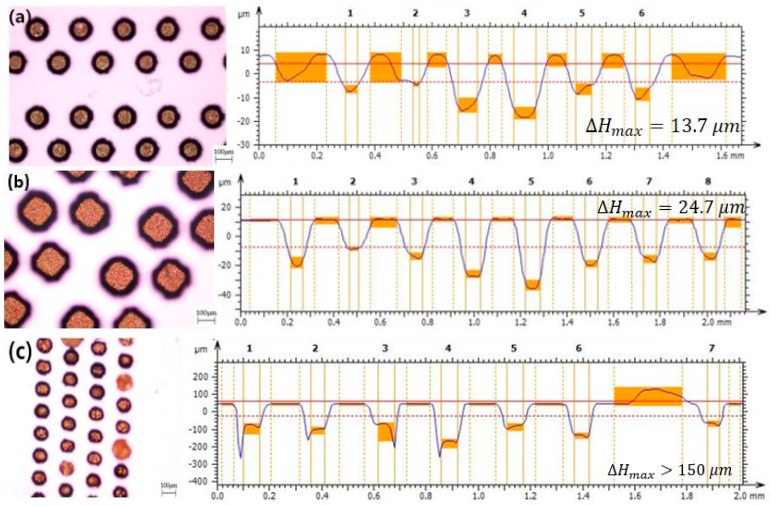
Top views and profile height diagram taken from the section with the greatest height difference using different shape holes using an optical microscope and a confocal displacement detector, respectively: (**a**) circle, (**b**) square, and (**c**) oval holes.

The experimental results are shown in Figure 7. Under the same experimental conditions, electroplated copper grown in round and square holes attained good uniformity, which was significantly better than the uniformity of oval holes, and they were less affected by current.

For the oval through holes, due to the different Cu^2+^ diffusion speeds in two directions, the reaction speeds of Cu^2+^ in different parts of the oval holes remained inconsistent. Because of the diffusion speed of Cu^2+^ toward the sides and corners being different, different concentrations were generated. We concluded that better uniformity could be obtained using a circular through via shape.

## 4. Fabrication of a 3D Inductor

**Figure 8:**
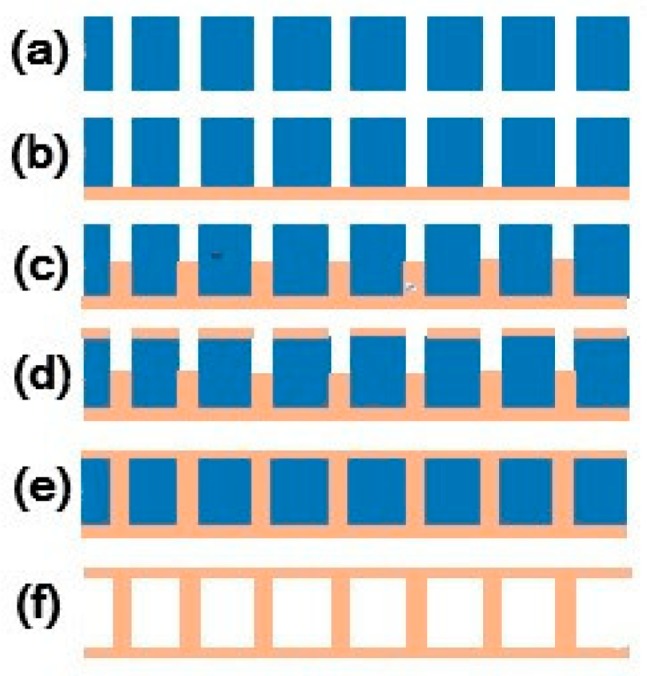
Three-dimensional (3D) inductor manufacturing steps: (**a**) TSVs were etched and a SiO_2_ insulation layer was deposited. (**b**) The adhesion layer (Cr, 30 nm) and seed layer (Cu, 300 nm) were sputtered and electroplated copper covered the TSVs. (**c**) Electroplated copper was grown in TSVs. (**d**) The copper layer was sputtered on the top side. (**e**) The TSVs were fully filled and (**f**) the silicon substrate was removed.

Based on the processing optimization and successful filling of 500 μm high-aspect-ratio TSVs, we processed a 1000 μm high and 100 μm diameter 3D solenoid inductor. The 3D micro-inductor processing steps are presented in Figure 8.

**Figure 9:**
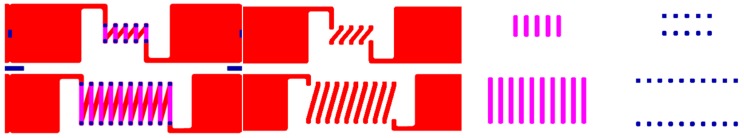
The design of the three-dimensional inductor.

**Figure 10:**
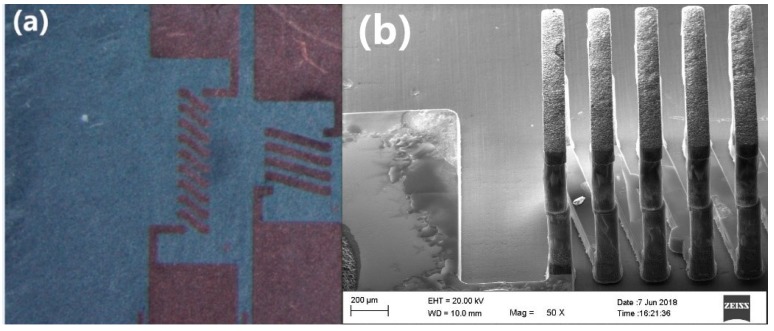
The silicon corn inductor (**a**) photographed by the peripheral microscope and (**b**) a detailed image photographed by a scanning electron microscope (SEM).

Figure 9 shows the design of the 3D inductor. After the through-vias were etched, the seed layer was sputtered on the surface and the electroplating process began. When the electroplated copper grew close to the upper surface, magnetron sputtering was performed to adhere the copper to the surface. Electroplating for the through-vias occurred until the upper surface was covered. After the electroplating process, we employed chemical mechanical polishing to remove the excessive copper on the surface, obtaining the 3D inductor structure and the silicon corn inductor as shown in Figure 10.

**Figure 11:**
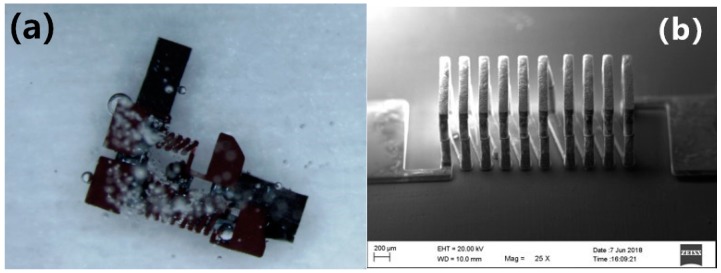
(**a**) 3D hollow inductor in tetramethylammonium hydroxide (TMAH) solution photographed by peripheral microscopy and (**b**) the hollow inductor after silicon removed, as photographed by SEM.

In order to obtain an air-core MEMS inductor, the silicon core inductor was immersed in 8% TMAH (Tetramethylammonium Hydroxide) solution for nearly 16 h and silicon was removed using the wet etching method. The final solenoid inductance is shown in Figure 11.

## 5. Conclusions

This study focused on the filling of high-aspect-ratio TSVs via electroplating copper. The influence of different experimental parameters, including current density, additive concentration, and different shapes of TSVs on the electroplating results, was analyzed.

Increasing current density improved the electroplating speed, but led to poor uniformity. The additive concentration needs to be maintained within a suitable range, since too low or too high a concentration affects the results. Regarding the influence of different shapes, through vias that were more circular in shaped produced more uniform results. Considering the combination of electroplating speed and uniformity, the optimized parameters for electroplating experiments were 1.5 A/dm^2^ current density and 1 mL/L additive concentration. Applying these parameters, the complete filling of the 500-μm-deep high-density TSVs with an aspect ratio of 10:1 was realized at the fastest electroplating speed, producing relatively good uniformity whose coefficient of variation is 0.19. Using high-aspect-ratio TSV electroplating and removing excess silicon with TMAH solution, a 1000-μm-high 3D air-core copper solenoid inductor with 10 turns was successfully fabricated, which is the tallest micro-inductor currently produced using micromachining. The measurement results show that when the frequency is 500 MHz, the inductance is 70 nH and the quality factor is 14.

## Figures and Tables

**Table 1:** The basic electroplating solution composition. (Applied in industry).

Electroplating Solution Composition	Concentration
Deionized water	850 mL/L
CuSO_4_·5H_2_O	220 g/L
H_2_SO_4_	38.5 mL/L
NaCl	0.165 g/L
Cupracid Ultra Make-up	10 mL/L
Cupracid Ultra A	0.5 mL/L
Cupracid Ultra B	0.5 mL/L

**Table 2:** Control variable experimental parameters.

Experiment Number	Current Density (A/dm^2^)	Additives (mL/L)	Through Vias Shapes
1	0.25	0.5	Circle
2	0.5	0.5	Circle
3	1	0.5	Circle
4	1.25	0.5	Circle
5	1.5	0.5	Circle
6	1.75	0.5	Circle
7	2	0.5	Circle
8	1	0.25	Circle
9	1	1	Circle
10	1	1.5	Circle
11	1	1	Circle
12	1	1	Oval
13	1	1	Square

**Table 3:** Electroplating experiment results.

Experiment Number	Electroplating Time (h)	Average Height (μm)	Average Growth Speed of Copper (μm/h)	The Most Height Difference of Vias/μm	Coefficient of Variation
1	>100	-	<5	-	-
2	72	481.7	6.69	14.5	0.12
3	50	473.2	9.45	16	0.14
4	27	482.4	17.87	18.4	0.17
5	19	471.2	24.8	23.9	0.19
6	17	478.8	28.16	43.2	0.26
7	8	-	>62.5	>500	0.41
8	55	-	-	>200	0.39
9	47	476.7	10.14	11.1	0.11
10	45	485.6	10.79	37.8	0.32
11	50	483.2	9.66	13.7	0.11
12	50	478.3	9.56	24.7	0.15
13	50	-	-	>150	0.37

## References

[1] Lu J.-Q. (2009). 3-D hyper integration and packaging technologies for micronano systems. Proc. IEEE.

[2] Gutmann R.J., Lu J.Q., Devarajan S., Zeng A.Y., Rose K. Wafer-level three-dimensional monolithic integration for heterogeneous silicon ICs. Proceedings of the Topical Meeting onSilicon Monolithic Integrated Circuits in RF Systems.

[3] Banerjee K., Souri S.J., Kapur P., Saraswat K.C. (2001). 3-D ICs: A novel chip design for improving deep-submicrometer interconnect performance and systems-on-chip integration. Proc. IEEE.

[4] Kettner P., Kim B., Pargfrieder S., Zhu S. New Technologies for advanced high density 3D packaging by using TSV process. Proceedings of the Electronic Packaging Technology & High Density Packaging.

[5] Lai X.H., Ding F., Xu Z.G., Wu W.G., Xu J., Hao Y.L. Suspended nanoscale solenoid metal inductor with tens-nH level inductance. Proceedings of the International Conference on MICRO Electro Mechanical Systems.

[6] Gallé W.P. (2012). MEMS-Based Fabrication of Power Electronics Components for Advanced Power Converters.

[7] Domann J.P., Chen C., Sepulveda A.E., Candler R.N., Carman G.P. (2018). Multiferroic Micro-Motors with Deterministic Single Input Control. arXiv.

[8] Xu L., Dixit P., Miao J., Pang J.H., Zhang X., Tu K.N., Preisser R. (2007). Through-wafer electroplated copper interconnect with ultrafine grains and high density of nanotwins. Appl. Phys. Lett..

[9] Tenno R., Pohjoranta A. (2008). An ALE Model for Prediction and Control of the Microvia Fill Process with Two Additives. J. Electrochem. Soc..

[10] Yan C.P. (2011). Studying on Key Process in TSV Technology. Ph.D. Thesis.

[11] Huang C. (2015). High Performance Through-Silicon-Via in 3D Interconnection (TSV).

[12] Chow E.M., Chandrasekaran V., Partridge A., Nishida T., Sheplak M., Quate C.F., Kenny T.W. (2002). Process compatible polysilicon-based electrical through-wafer interconnects in silicon substrates. J. Microelectromech. Syst..

[13] Moffat T.P., Wheeler D., Huber W.H., Josell D. (2001). Superconformal Electrodeposition of Copper [Electrochemical and Solid-State Letters, 4, C26 (2001)]. Electrochem. Solid-State Lett..

[14] Wei H., Shi K. (2014). Numerical study of TSV Copper Deposition with Multi-additives. Electron. Process. Technol..

[15] Yi’nan L.I., Jian C.A.I., Dejun W.A.N.G., Qian W.A.N.G., Tiwei W.E.I. (2012). Optimizing Copper Filling Process For Through Silicon Via (TSV). Equip. Electron. Prod. Maruf..

[16] Lee J., Lee J., Bae J., Bang W., Hong K., Lee M.H., Pyo S.G., Kim S., Kim J.G. (2006). An Approach to the Development of Organic Additives for Electrodeposition of Narrow Copper Interconnects. Surgery.

[17] Wang F., Zeng P., Wang Y., Ren X., Xiao H., Zhu W. (2017). High-speed and high-quality TSV filling with the direct ultrasonic agitation for copper electrodeposition. Microelectron. Eng..

[18] Tian Q., Cai J., Zheng J., Zhou C., Li J., Zhu W. (2017). Copper Pulse-Reverse Current Electrodeposition to Fill Blind Vias for 3-D TSV Integration. IEEE Trans. Compon. Packag. Manuf. Technol..

[19] Li Y., Cao H., Feng X., Ling H., Li M., Sun J. Effect of different pretreatments on through silicon via copper filling. Proceedings of the International Conference on Electronic Packaging Technology.

[20] Xu C., Wang X., Wang Y., Xu M., Hu C., Liu S. Void free filling of TSV vias by bottom up copper electroplating for wafer level MEMS vacuum packaging. Proceedings of the 2012 International Conference on Electronic Packaging Technology & High Pachaging.

[21] Chuang H.C., Li H.F., Lin Y.S., Lin Y.H., Huang C.S. (2013). The development of an atom chip with through silicon vias for an ultra-high-vacuum cell. J. Micromech. Microeng..

[22] Xiao H., Wang F., Wang Y., He H., Zhu W. (2017). Effect of Ultrasound on Copper Filling of High Aspect Ratio Through-Silicon Via (TSV). J. Electrochem. Soc..

[23] Roh M.H., Lee J.H., Kim W., Pil Jung J. (2013). Cu filling of TSV using various current forms for three-dimensional packaging application. Solder. Surf. Mt. Technol..

[24] Meng W., Guan Y., Zeng Q., Chen J., Jin Y. Fabrication process of a triple-layer stacked TSV interposer for switch matrix consisting of eight RF chips. Proceedings of the Electronics Packaging Technology Conference.

[25] Sun J.J., Kondo K., Okamura T. High-Aspect-Ratio Copper Via Filling Used for Three-Dimensional Chip Stacking. Proceedings of the Electronic Components and Technology Conference.

[26] Gambino J.P., Adderly S.A., Knickerbocker J.U. (2015). An Overview of Through-Silicon-Via Technology and Manufacturing Challenges.

[27] Wu H. (2013). The Study on Copper Electro-Deposition in Through Silicon Vias.

[28] Choi E.H., Lee Y.S., Rha S.K. (2012). Effects of Current Density and Organic Additives on via Copper Electroplating for 3D Packaging. Korean J. Mater. Res..

[29] Wang Z., Wang H., Cheng P., Ding G., Zhao X. (2014). Simultaneous filling of through silicon vias (TSVs) with different aspect ratios using multi-step direct current density. J. Micromech. Microeng..

